# Clinical significance of prognostic nutritional index (PNI)-monocyte-to-lymphocyte ratio (MLR)-platelet (PLT) score on postoperative outcomes in non-metastatic clear cell renal cell carcinoma

**DOI:** 10.1186/s12893-023-02001-x

**Published:** 2023-05-10

**Authors:** Wenming Ren, Hao Zhang, Li Cheng, Yu Zhang, Chenglin Yang, Liang Nie, Congcong Yang, Peng Yao, Jie Han, Dong Zhuo

**Affiliations:** Department of Urology, the First Affiliated Hospital of Wannan Medical College (Yijishan Hospital of Wannan Medical College), Wuhu, Anhui 241000 China

**Keywords:** PNI-MLR-PLT score, Non-metastatic, Clear cell renal cell carcinoma (ccRCC), Prognostic indicator

## Abstract

**Background:**

Prognositic nutritional index (PNI), monocyte-to-lymphocyte ratio (MLR) and platelet (PLT) are associated with tumor survival in many human malignancies. Whereas, no study combined PNI-MLR-PLT score and indicated its predictive significance on the prognosis of patients with non-metastatic clear cell renal cell carcinoma (ccRCC).

**Methods:**

In this study, we retrospectively collected the clinicopathological characteristics and prognostic data from 164 cases of non-metastatic ccRCC and aimed to determine the clinical significance of PNI-MLR-PLT score on patients’ outcomes after surgery. The optimal cut-off values of PNI (PNI > 47.40 vs PNI < 47.40), MLR (MLR > 0.31 vs MLR < 0.31) and PLT (PLT > 245 vs PLT < 245) were identified with relative operating characteristic (ROC) curve analysis. The PNI-MLR-PLT score system was established by the value of three indexes, each indication was assigned a score of 0 or 1. Overall survival (OS) and metastasis-free survival (MFS) were analyzed using Kaplan–Meier estimate and Cox regression models.

**Results:**

The mean follow-up period was 85.67 months. Eight (5.0%) patients died, 4 (2.0%) relapsed, and 7 (4.0%) developed metastasis after surgery. The 3-year OS and MFS rates were 98.2% and 97.6%, and the 5-year OS and MFS rates were both 90.2%. Our results suggested that PNI-MLR-PLT score negatively correlated with pathological T stage and tumor grade. Survival outcomes revealed that lower PNI-MLR-PLT score is associated with inferior OS (*P* < 0.001) and MFS (*P* < 0.001) after surgery. Subgroup analysis regarding pathological T stage, tumor grade and surgical modalities obtained consistent results. univariable and multivariable Cox analysis showed that high PNI-MLR-PLT score was the independent protective factor of tumor survival in non-metastatic ccRCC patients.

**Conclusions:**

Our data suggested that PNI-MLR-PLT score could serve as a promising independent prognostic factor in patients with non-metastatic ccRCC.

## Introduction

Renal cell carcinoma (RCC) represents the third most frequent cancer in urology. The most common subtype of RCC is clear cell renal cell carcinoma (ccRCC) [1]. Radical or partial nephrectomy are now the standard treatment for localized lesions [2]. Despite the anatomical tumor excision achieved by surgery, tumor recurrence or metastasis occurred in about one third of RCC patients, which have dismal 5-year survival rates [3, 4]. The effective prognostic indicators are urgently needed in clinical. At present, most of the prognostic models for ccRCC patients are established by combining gene expression profile, which is costly and sample inaccessible [5, 6]. Current studies have shown that preoperative nutritional index, namely prognostic nutritional index (PNI), is associated with postoperative survival outcomes in human cancers, including gastric [7], esophageal [8] and lung cancers [9]. Furthermore, the clinical significance of monocyte-to-lymphocyte ratio (MLR) and platelet (PLT) has also been confirmed [10–13]. In this study, we developed a novel model containing PNI, MLR and PLT and investigated its predictive value of PNI-MLR-PLT score on the prognosis of patients with non-metastatic ccRCC.

## Materials and methods

### Patients’ data

From September 2011 to August 2016, 204 patients with non-metastatic ccRCC who underwent either radical or partial nephrectomy in our center were reviewed. Then 40 cases were excluded due to the incomplete clinical or prognostic information. Pathological stage and tumor grade of each patient were determined by one and the same pathologist based on the 8^th^ American Joint Committee on Cancer (AJCC) tumor-node-metastasis (TNM) staging and the 2016 WHO/ISUP G grading system for ccRCC, respectively. Demographic and laboratory test results were retrospectively recorded using our medical system. PNI was calculated as albumin (g/L) + 5 * total number of peripheral blood lymphocytes (L). MLR was defined as monocyte-to-lymphocyte ratio. Diabetes mellitus (DM) was diagnosed as fasting (no food intake for 8 h) venous glucose >= 7.0 mmol/L; venous glucose >= 11.1 mmol/L after 2 h of oral glucose tolerance test OGTT; non-fasting glycated hemoglobin HbA1C test >= 6.5% or the requirement of oral hypoglycemic medications and/or insulin. Systolic blood pressure >= 140mmHg and/or a diastolic blood pressure >= 90mmHg without antihypertensive treatment were considered as hypertension. Anaemia was defined as serum hemoglobin ≤130g/dl in adult males and ≤120g/dl in adult females. Patients received physical reexamination including blood tests, ultrasound or CT at least once a year after surgery, and the survival data were recorded. The latest follow-up date is due to August 31, 2021. The time from nephrectomy to death was defined as overall survival (OS). The interval between post-nephrectomy and the presence of imaging or histological evidence of distant metastases was defined as metastasis-free survival (MFS).

### Statistical and analysis methods

The data were analyzed using SPSS 19.0 statistical software (IBM SPSS INC., Chicago, USA). The best cut-off values of PNI, MLR and PLT were determined using the relative operating characteristic (ROC) curve according to the Youden Index. The PNI-MLR-PLT score was assigned as the sum scores of the three indexes, with each index scoring 0 or 1. Kaplan-Meier analysis was used to reveal the survival rates between patient groups. Continuous variables with normal distribution are presented as means ± standard deviations (SD). Non‐normal continuous variables are presented as medians (interquartile ranges). Categorical variables are presented as numbers (%), and the correlations between categorical variables were analyzed using chi-square test. Univariable and multivariable Cox proportional risk regression models were used to investigate the hazard ratios (HRs) of significant risk predictors with respect to OS and MFS. Area under ROC curve (AUC) value was used to access the discriminant ability of various parameters. The difference was regarded statistically significant when *P* <0.05.

## Results

### Baseline characteristics

**Fig. 1:**
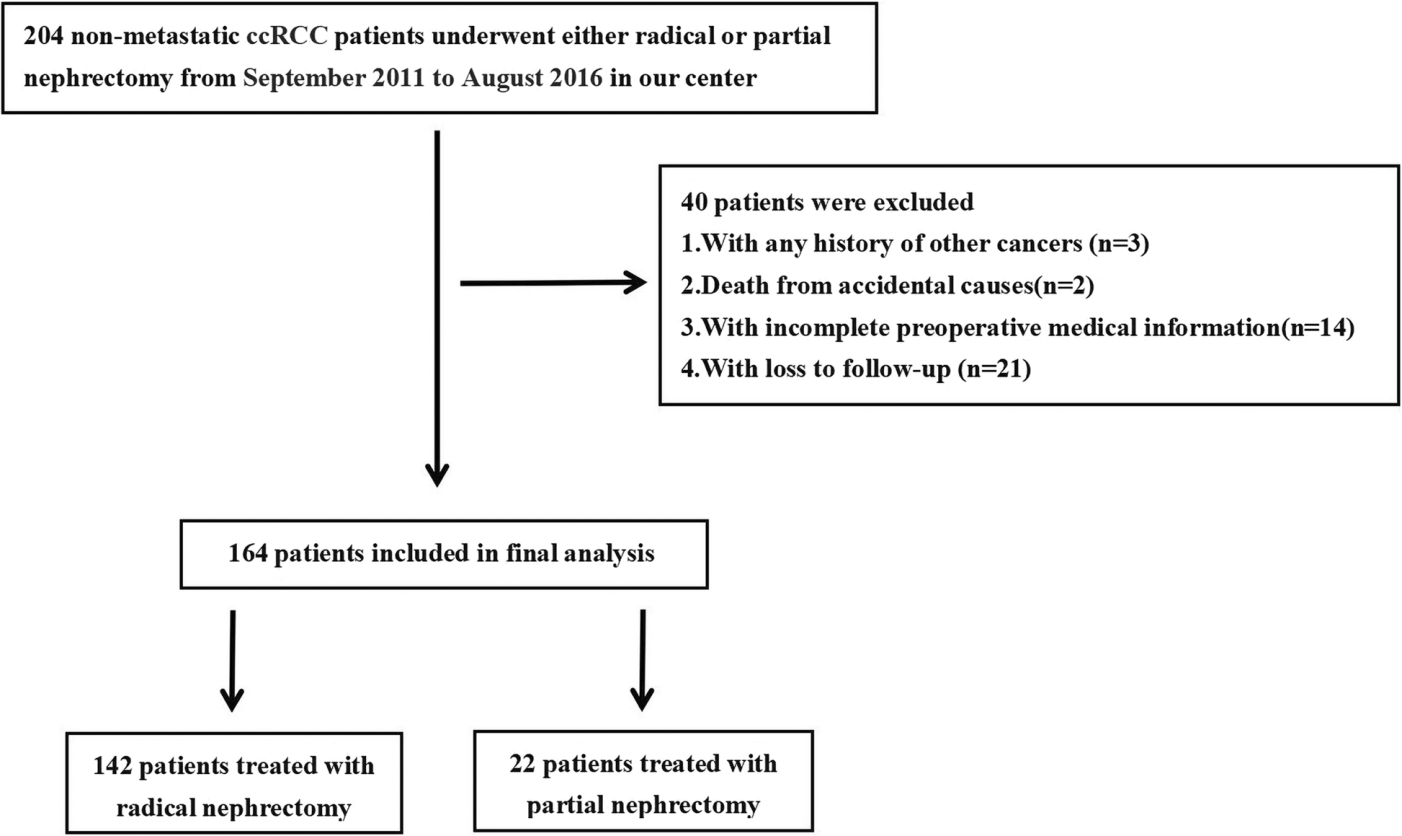
The flowchart of patients enrolled in this study

A total of 164 of 204 non-metastatic ccRCC patients were enrolled in this study (Fig. 1). Tables 1 and 2 described the demographic and clinicopathological data of these patients. As depicted, this study included 99 (60%) male patients and 65 (40%) female patients with an average age at surgery of 56.72 ± 11.78 years. The age distribution was as follows: 45 (27%) patients were >= 65 years and 119 (73%) patients were < 65 years. Of these patients, 24 (15%) underwent partial nephrectomy and 140 (85%) had radical nephrectomy. The pathological stages included T1, T2, T3 and T4 in 134 (82%), 17 (10%), 7 (4.0%) and 3 (2.0%) cases, respectively. In addition, 99 (60%), 45 (27%), 7 (4.0%) and 6 (4.0%) patients presented with grade 1, grade 2, grade 3 and grade 4. The mean follow-up duration was 85.67 months (range, 6-121 months). By our follow-up deadline, 8 (5.0%) patients died, 4 (2.0%) relapsed, and 7 (4.0%) developed metastasis. The 3-year OS and MFS rates were 98.2% and 97.6%, respectively. The 5-year OS and MFS rates were both 90.2%.

**Table 1:** Clinicopathological data of patients with non-metastatic ccRCC

Parameter	Total (*n* = 164)
Gender (male/female)	99 (60%)/65 (40%)
Age, years (≥ 65/ < 65)	45 (27%)/119 (73%)
Drinking (yes/no)	23 (14%)/141 (86%)
Smoking (yes/no)	51 (31%)/113 (69%)
DM (yes/no)	16 (10%)/148 (90%)
Hypertension (yes/no)	68 (41%)/96 (59%)
Anemia (yes/no)	55 (34%)/109 (66%)
Hypoalbuminemia (yes/no)	10 (6.0%)/154 (94%)
Surgical approach (Partial nephrectomy/ Radical nephrectomy)	22 (13%)/142 (87%)
Renal dysfunction (yes/no)	8 (5.0%)/156 (95%)
Tumor number (> = 2/1)	4 (2.0%)/160 (98%)
Site (left/right)	84 (51%)/80 (49%)
Pathologic T stage (T1/T2/T3/T4)	134 (82%)/17 (10%)/10 (6%)/3 (2.0%)
G grade (1/2/3/4)	99 (60%)/45 (27%)/7 (4.0%)/6 (4.0%)
Tumor necrosis (yes/no)	6 (4.0%)/158 (96%)
Tumor hemorrhage	12 (7.0%)/152 (93%)
Lymphatic and microvascular infiltration	7 (4.0%)/157 (96%)
PNI (> 47.40/ < 47.40)	119 (73%)/45 (27%)
MLR (> 0.31/ < 0.31)	57 (35%)/107 (65%)
PLT (> 245/ < 245)	16 (10%)/148 (90%)
PMP score (0/1/2/3)	7 (4.0%)/24 (15%)/49 (30%)/84 (51%)

*ccRCC* Clear cell renal cell carcinoma, *DM* Diabetes mellitus, *PNI* Prognostic Nutritional Index, *MLR* Monocyte-to-lymphocyte ratio, *PLT* Platelet, *PMP score* PNI-MLR-PLT score

**Table 2:** Clinical and laboratory data in 164 patients with non-metastatic RCC

Parameter	Total (*n* = 164)
Age, years	56.72 ± 11.78
Tumor size, cm	4.00(3.00, 5.00)
Serum creatinine, mg/dl	73.60(61.63, 88.28)
BUN, mg/dl	5.81(4.84, 6.81)
ALT, U/l	18.50(13.00, 30.00)
AST, U/l	18.00(15.00, 23.00)
Hemoglobin, g/dl	129.16 ± 17.87
MLR	0.27(0.21, 0.36)
PLT	172.43 ± 64.14
PNI	50.78 ± 6.17

*ccRCC* Clear cell renal cell carcinoma, *BUN* Urea nitrogen, *ALT* Alanine aminotransferase, *AST* Glutamate aminotransferase, *MLR* Monocyte-to-lymphocyte ratio, *PLT* Platelet, *PNI* Prognostic nutritional index

**Fig. 2:**
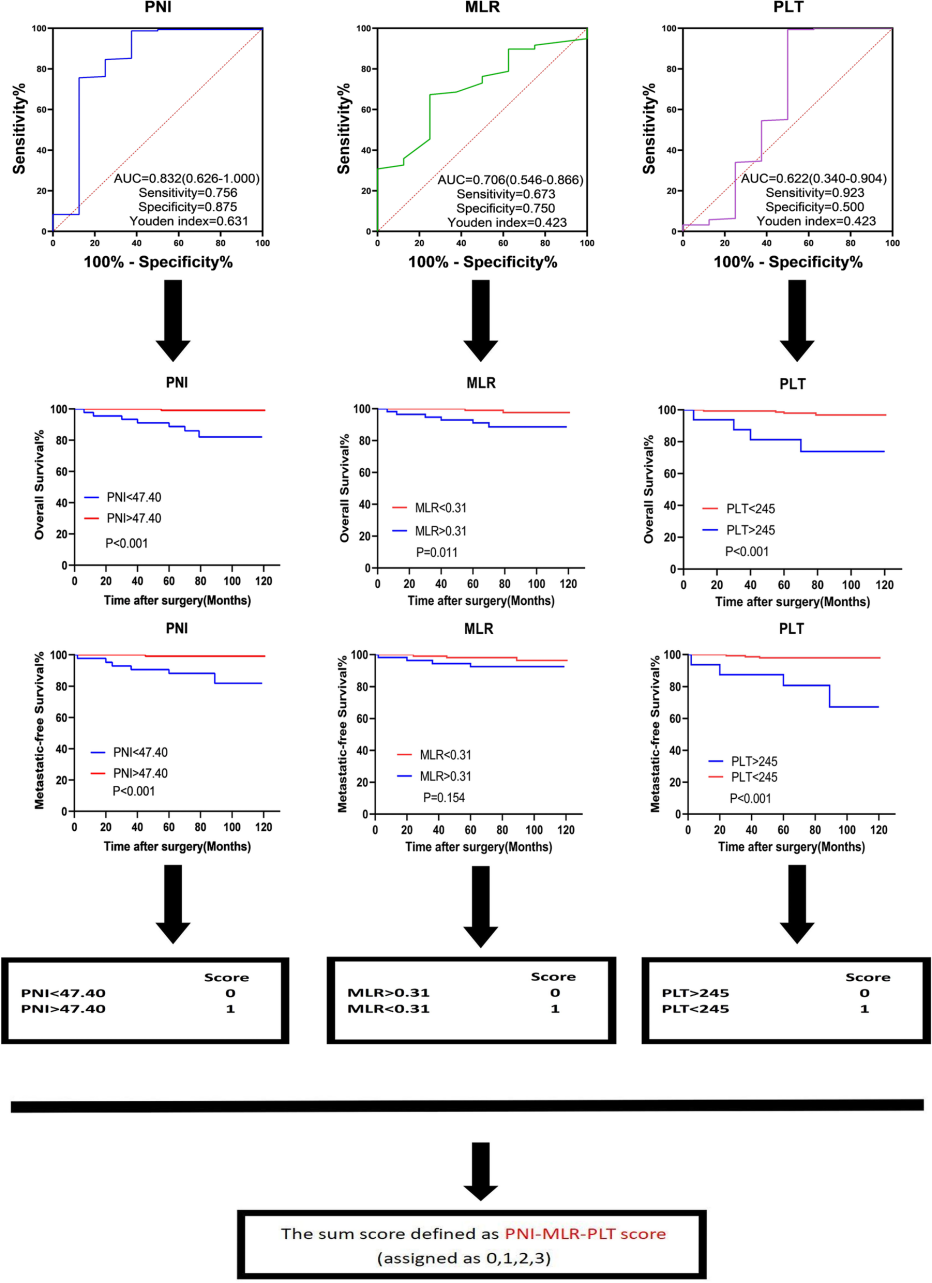
Flowchart showing PNI-MLR-PLT score

The best cut-off values of PNI, MLR and PLT were determined by ROC curves (Fig. 2). Kaplan-Meier curves revealed the discrepant OS and MFS rates between high and low PNI (PNI>47.40 vs PNI< 47.40), MLR (MLR >0.31 vs MLR <0.31) and PLT (PLT >245 vs PLT <245) patient groups (Fig. 2).

**Fig. 3:**
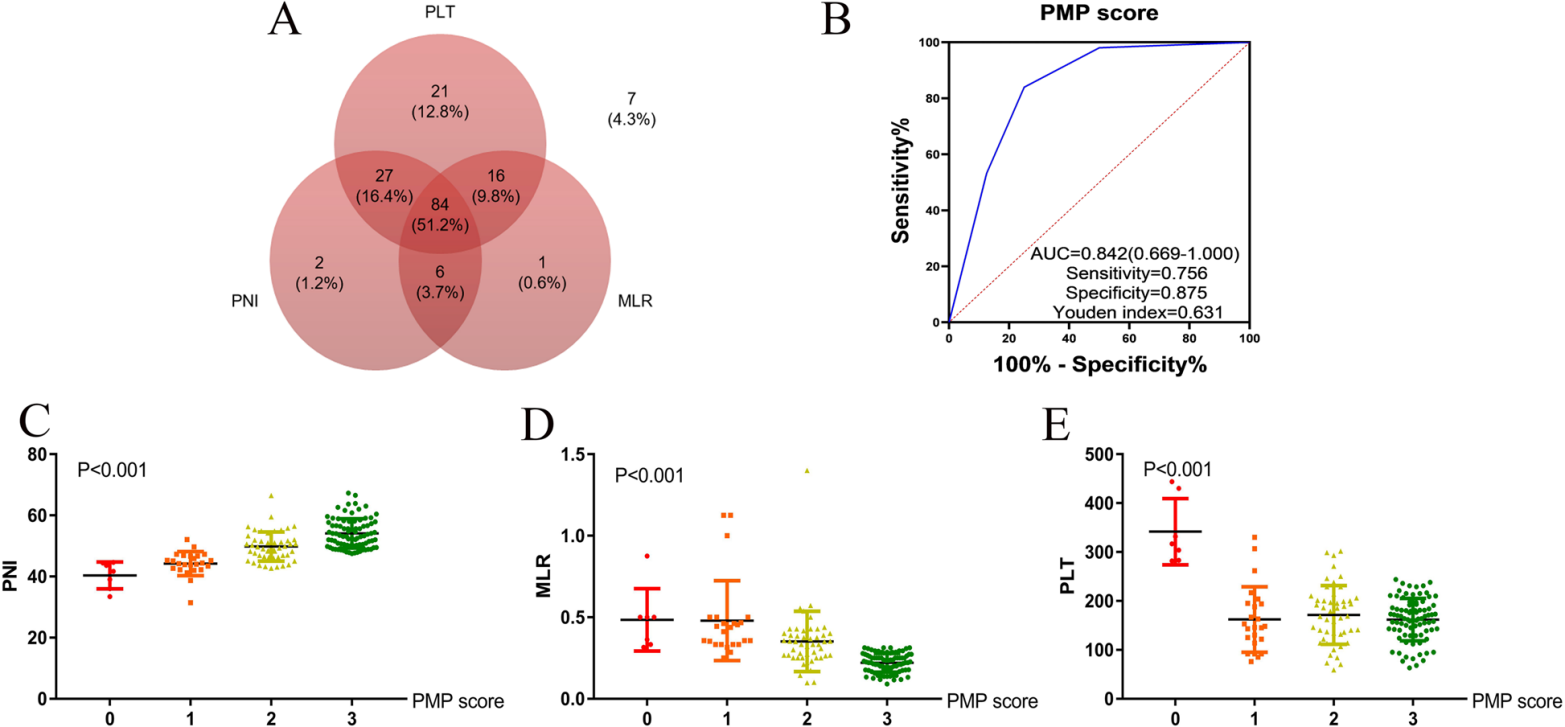
The Venn diagram of PNI, MLR and PLT (**A**). ROC curve of PNI-MLR-PLT score for OS of non-metastatic ccRCC patients (**B**). The distribution of PNI (**C**), MLR (**D**), and PLT (**E**) according to PNI-MLR-PLT score, respectively. PMP score: PNI-MLR-PLT score

The PNI-MLR-PLT score for each patient was calculated by the sum scores of the three assigned indicators (Fig. 2). As shown in the Venn diagram, 7 (4.0%) patients had a PNI-MLR-PLT score of 0, 24 (15%) patients had a score of 1, 49 (30%) patients had a score of 2, and 84 (51%) patients had a score of 3 (Fig. 3A).

### Patient characteristics and clinical outcomes based on PNI-MLR-PLT score

Table 3 showed diverse clinical characteristics or laboratory variables of these patients according to PNI-MLR-PLT score and their correlations.

**Table 3:** Baseline characteristics of patients with non-metastatic RCC according to PMP score

Parameters	PMP score				
	0 (*n* = 7)	1 (*n* = 24)	2 (*n* = 49)	3 (*n* = 84)	*P* value
Gender					0.728
Male (*n* = 99)	4 (2.0%)	17 (10%)	29 (17%)	49 (30%)	
Female (*n* = 65)	3 (2.0%)	7 (4.0%)	20 (12%)	35 (21%)	
Age (years)					0.051
< 65 (*n* = 119)	3 (2.0%)	14 (9.0%)	35 (21%)	67 (41%)	
> 65 (*n* = 45)	4 (2.0%)	10 (6.0%)	14 (9.0%)	17 (10%)	
Smoking					0.065
Yes (*n* = 51)	2 (1.0%)	13 (8.0%)	12 (7.0%)	24 (15%)	
No (*n* = 113)	5 (3.0%)	11 (7.0%)	37 (23%)	60 (37%)	
Drinking					0.774
Yes (*n* = 23)	1 (1.0%)	5 (3.0%)	6 (4.0%)	11 (7.0%)	
No (*n* = 141)	6 (4.0%)	19 (12%)	43 (26%)	73 (45%)	
Hypertension					0.603
Yes (*n* = 68)	4 (2.0%)	11 (7.0%)	22 (13%)	31 (19%)	
No (*n* = 96)	3 (2.0%)	13 (8.0%)	27 (16%)	53 (32%)	
DM					0.410
Yes (*n* = 16)	0 (0)	4 (2.0%)	3 (2.0%)	9 (5.0%)	
No (*n* = 148)	7 (4.0%)	20 (12%)	46 (28%)	75 (46%)	
Anemia					< 0.001
Yes (*n* = 55)	7 (4.0%)	14 (9.0%)	16 (10%)	18 (11%)	
No (*n* = 109)	0 (0)	10 (6.0%)	33 (20%)	66 (40%)	
Renal dysfunction					0.033
Yes (*n* = 8)	0 (0)	4 (2.0%)	2 (1.0%)	2 (1.0%)	
No (*n* = 156)	7 (4.0%)	20 (12%)	47 (29%)	82 (50%)	
ALT					0.387
> 13.5 (*n* = 117)	3 (2.0%)	17 (10%)	35 (21%)	62 (38%)	
< 13.5 (*n* = 47)	4 (2.0%)	7 (4.0%)	14 (9.0%)	22 (13%)	
AST					0.652
> 19.5 (*n* = 68)	2 (1.0%)	8 (5.0%)	20 (12%)	38 (23%)	
< 19.5 (*n* = 96)	5 (3.0)	16 (10%)	29 (18%)	46 (28%)	
BUN					0.171
> 7.325 (*n* = 27)	2 (1.0%)	7 (4.0%)	5 (3.0%)	13 (8.0%)	
< 7.325 (*n* = 137)	5 (3.0%)	17 (10%)	44 (27%)	71 (43%)	
PLT					
> 245 (*n* = 16)	7 (4.0%)	3 (2.0%)	6 (4.0%)	0 (0)	< 0.001
< 245 (*n* = 148)	0 (0)	21 (13%)	43 (26%)	84 (51%)	
MLR					
> 0.31 (*n* = 57)	7 (4.0%)	23 (14%)	27 (16%)	0 (0)	< 0.001
< 0.31 (*n* = 107)	0 (0)	1 (1.0%)	22 (13%)	84 (51%)	
PNI					
> 47.40 (*n* = 119)	0 (0)	2 (1.0%)	33 (20%)	84 (51%)	< 0.001
< 47.40 (*n* = 45)	7 (4.0%)	22 (13%)	16 (10%)	0 (0)	
Tumor number					0.739
> = 2 (*n* = 4)	0 (0)	0 (0)	1 (1.0%)	3 (2.0%)	
< 2 (*n* = 160)	7 (4.0%)	24 (15%)	48 (29%)	81 (49%)	
Site					0.219
Left (*n* = 84)	2 (1.0%)	9 (6.0%)	25 (15%)	48 (30%)	
Right (*n* = 80)	5 (3.0%)	15 (9.0%)	24 (15%)	36 (22%)	
Tumor size (cm)					< 0.001
> 7.3 (*n* = 13)	3 (2.0%)	4 (2.0%)	5 (3.0%)	1 (1.0%)	
< 7.3 (*n* = 151)	4 (2.0%)	20 (12%)	44 (27%)	83 (51%)	
Grade					< 0.001
1 (*n* = 99)	3 (2.0%)	12 (7.0%)	28 (17%)	56 (34%)	
2 (*n* = 45)	0 (0)	7 (4.0%)	16 (10%)	22 (13%)	
3 (*n* = 7)	1 (1.0%)	2 (1.0%)	2 (1.0%)	2 (1.0%)	
4 (*n* = 6)	3 (2.0%)	2 (1.0%)	1 (1.0%)	0 (0)	
T stage					< 0.001
1 (*n* = 134)	2 (1.0%)	14 (9.0%)	41 (25%)	77 (47%)	
2 (*n* = 17)	3 (2.0%)	6 (4.0%)	6 (4.0%)	2 (1.0%)	
3 (*n* = 10)	1 (1.0%)	2 (1.0%)	2 (1.0%)	5 (3.0%)	
4 (*n* = 3)	1 (1.0%)	2 (1.0%)	0 (0)	0 (0)	
Tumor hemorrhage					0.781
Yes (*n* = 12)	0 (0)	1 (1.0%)	4 (2.0%)	7 (4.0%)	
No (*n* = 152)	7 (4.0%)	23 (14%)	45 (27%)	77 (47%)	
Tumor necrosis					0.424
Yes (*n* = 6)	0 (0)	0 (0)	1 (1.0%)	5 (3.0%)	
No (*n* = 158)	7 (4.0%)	24 (15%)	48 (30%)	79 (48%)	
Lymphatic and microvascular infiltration					< 0.001
Yes (*n* = 7)	1 (1.0%)	4 (2.0%)	0 (0)	2 (1.0%)	
No (*n* = 157)	6 (4.0%)	20 (12%)	49 (30%)	82 (50%)	

*ccRCC* Clear cell renal cell carcinoma, *DM* Diabetes mellitus, *BUN* Urea nitrogen, *ALT* Alanine aminotransferase, *AST* Glutamate aminotransferase, *MLR* Monocyte-to-lymphocyte ratio, *PLT* Platelet, *PNI* Prognostic nutritional index, *PMP score* PNI-MLR-PLT score

**Fig. 4:**
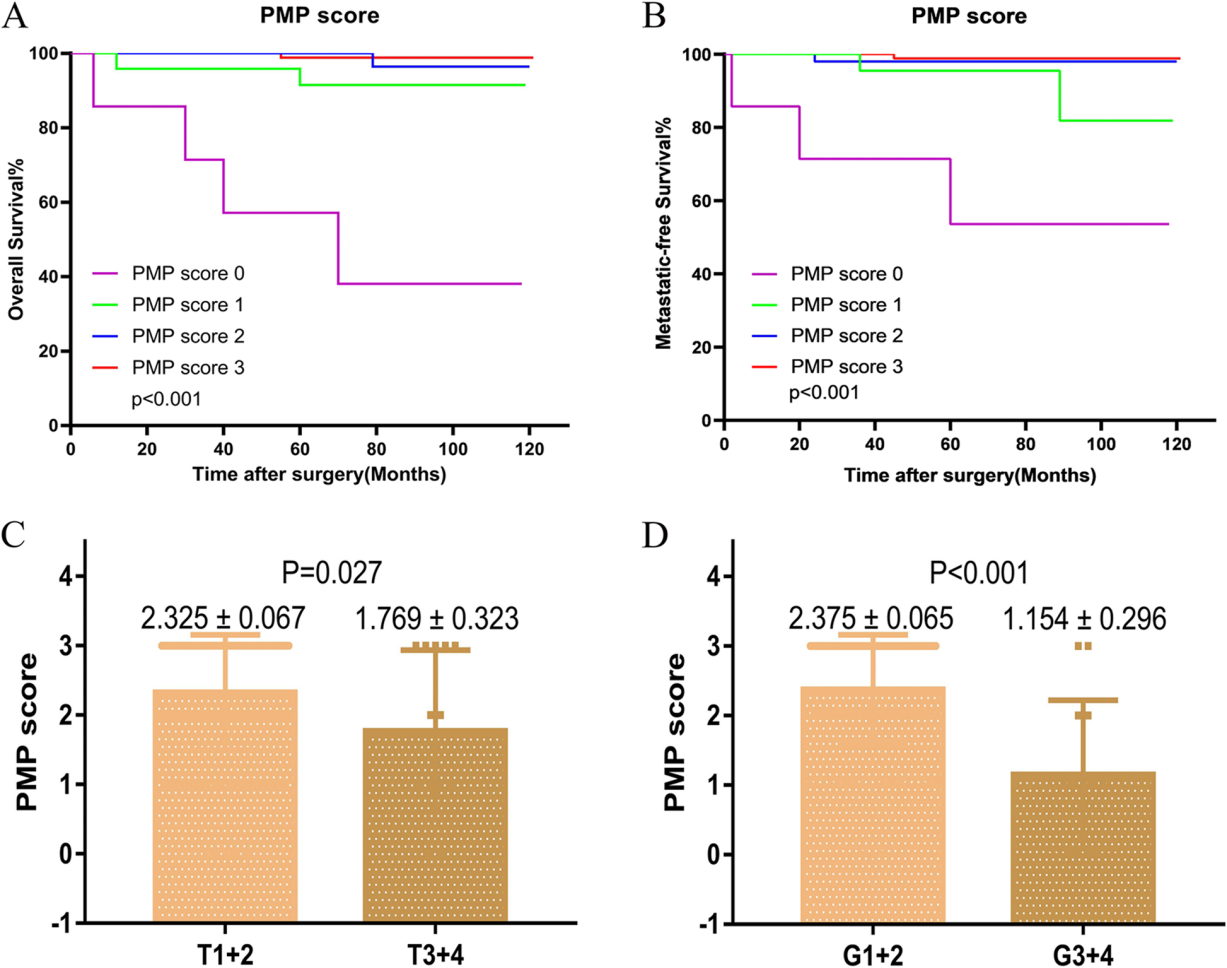
Kaplan–Meier analysis for overall survival (OS) (**A**) and metastatic-free survival (MFS) (**B**) in ccRCC patients based on PNI-MLR-PLT score. The distribution of PNI-MLR-PLT score according to pathologic T stage (**C**) and tumor grade (**D**), respectively. PMP score: PNI-MLR-PLT score

As a result, PNI-MLR-PLT score was significantly correlated with anemia, renal dysfunction, PLT, MLR, PNI, tumor size, pathologic T stage and tumor grade, lymphatic and microvascular infiltration (*P<*0.05). Figure 3B showed the ROC curve of PNI-MLR-PLT score for OS of non-metastatic ccRCC patients.PNI value increased with the rise of PNI-MLR-PLT score, while MLR and PLT values declined in ccRCC patients. Figure 3C-E showed the correlation between PNI-MLR-PLT score and the three indexes (all *P<*0.001). In addition, Kaplan-Meier analysis showed the discrepant survival outcomes among patients with different PNI-MLR-PLT score. The higher PNI-MLR-PLT score patient groups achieved significantly superior OS (*P<* 0.001) and MFS (*P<*0.001) than those with lower score (Fig. 4A and B). Moreover, as the Fig. 4C and D indicated, high PNI-MLR-PLT score was linked with lower pathological T stage and tumor grade.

**Fig. 5:**
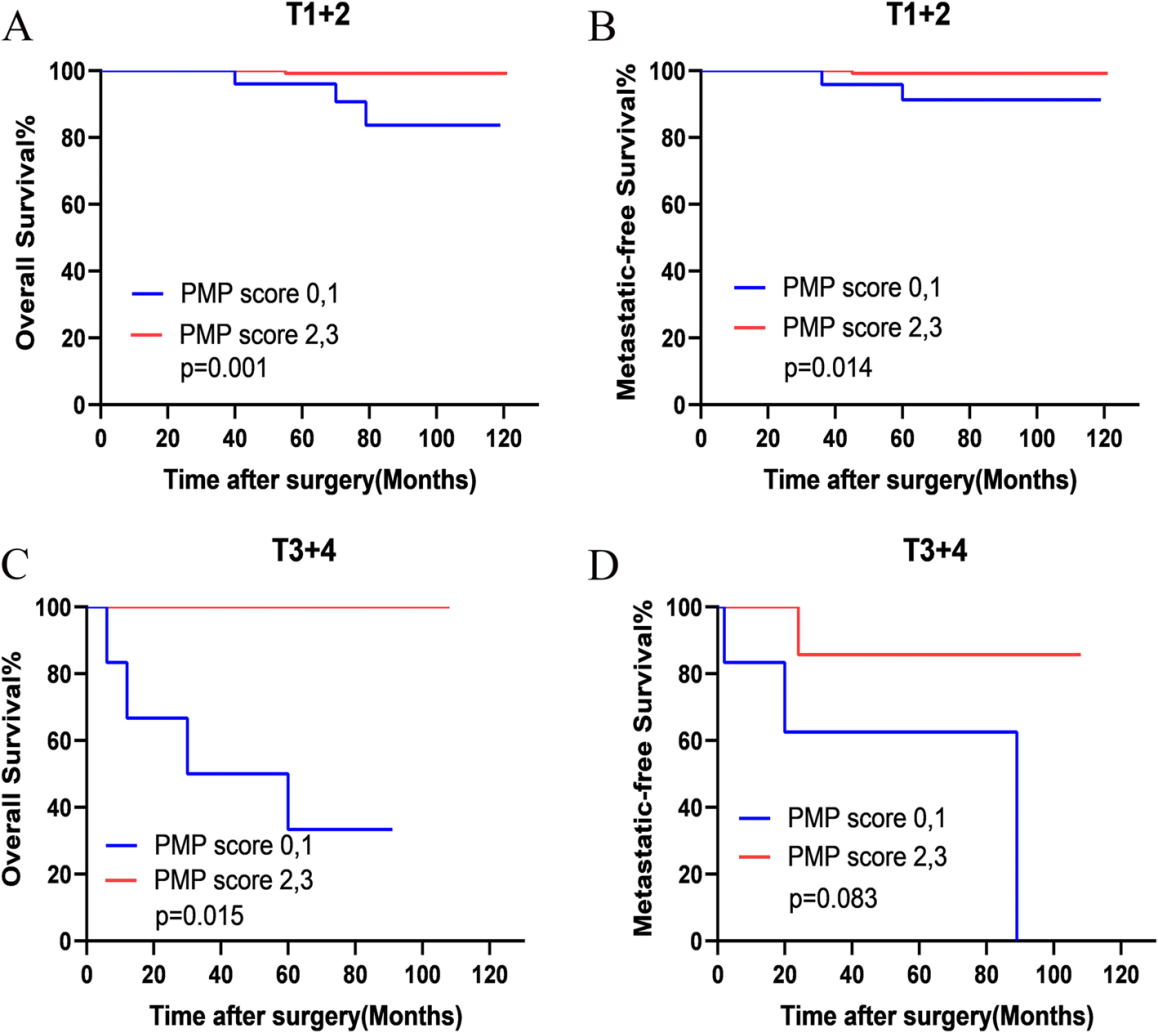
Stratified Kaplan–Meier analysis regarding pathologic T stage for overall survival (OS) (**A** and **C**) and metastatic-free survival (MFS) (**B** and **D**) of patients with high and low risk of PNI-MLR-PLT scores. PMP score: PNI-MLR-PLT score

We further divided these patients into two groups: low-risk group (scores 2 and 3) and high-risk group (scores 0 and 1). Then, a stratified analysis regarding T stage and tumor grade was performed. As expected, the pathological T stage (pT1+2 and pT3+4) subgroup analysis also indicated that the higher PNI-MLR-PLT score patients had better OS than those with lower PNI-MLR-PLT score in pT1+2 subgroup (pT1+2 group: OS (*P =* 0.001) and MFS (*P =* 0.014); pT3+4 group: OS (*P =* 0.015) and MFS (*P =* 0.083)) (Fig. 5A-D).

**Fig. 6:**
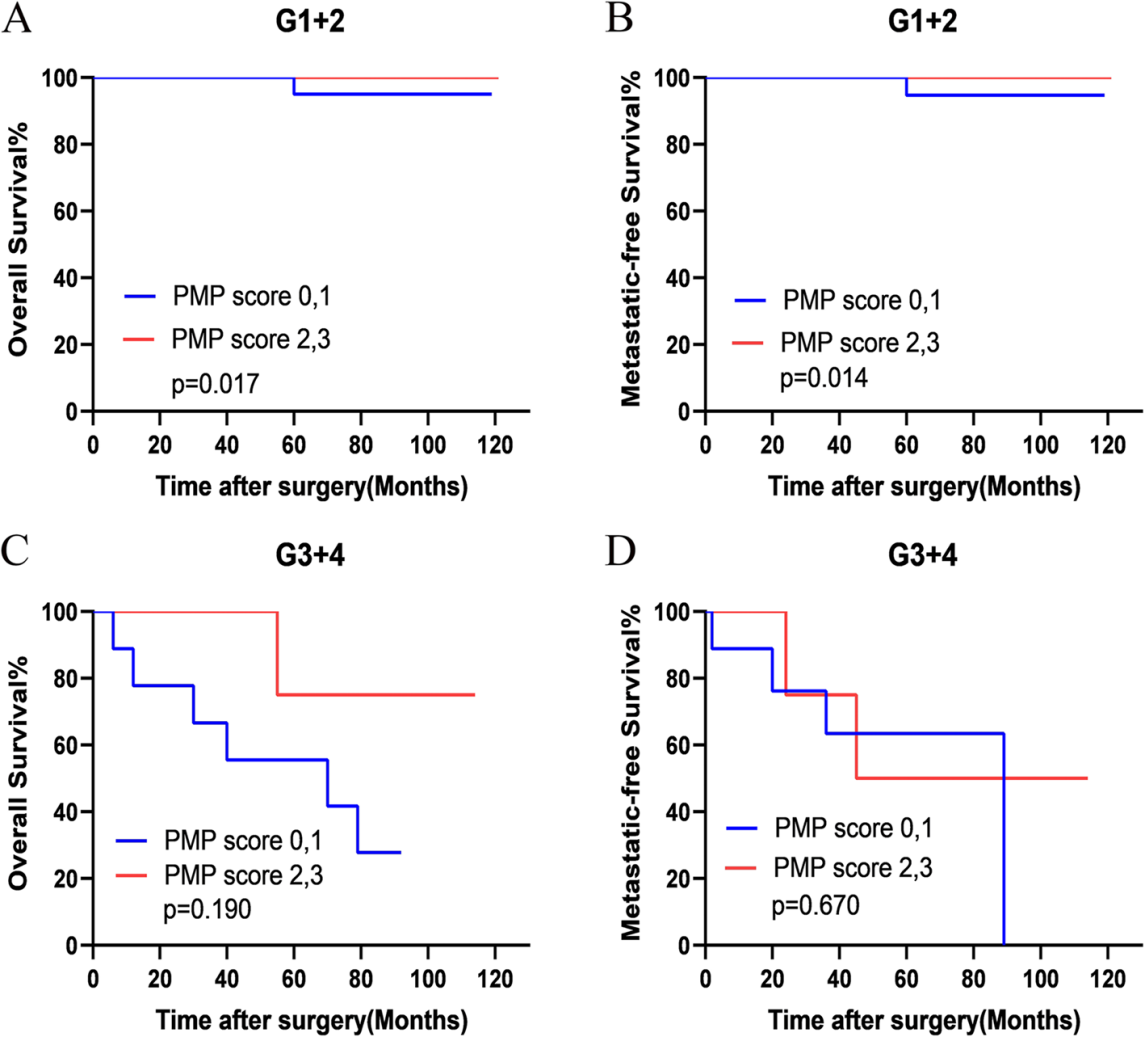
Stratified Kaplan–Meier analysis regarding pathologic tumor grade for overall survival (OS) (**A** and **C**) and metastatic-free survival (MFS) (**B** and **D**) of patients with high and low risk of PNI-MLR-PLT scores. PMP score: PNI-MLR-PLT score

Besides, subgroup analysis based on tumor grade (G1+2 and G3+4) also showed that patients with higher PNI-MLR-PLT score in the G1+2 group had better clinical outcomes (G1+2: OS (*P =*0.017) and MFS (*P =*0.014); G3+4: OS (*P =*0.190) and MFS (*P =*0.670) (Fig. 6A-D).

**Fig. 7:**
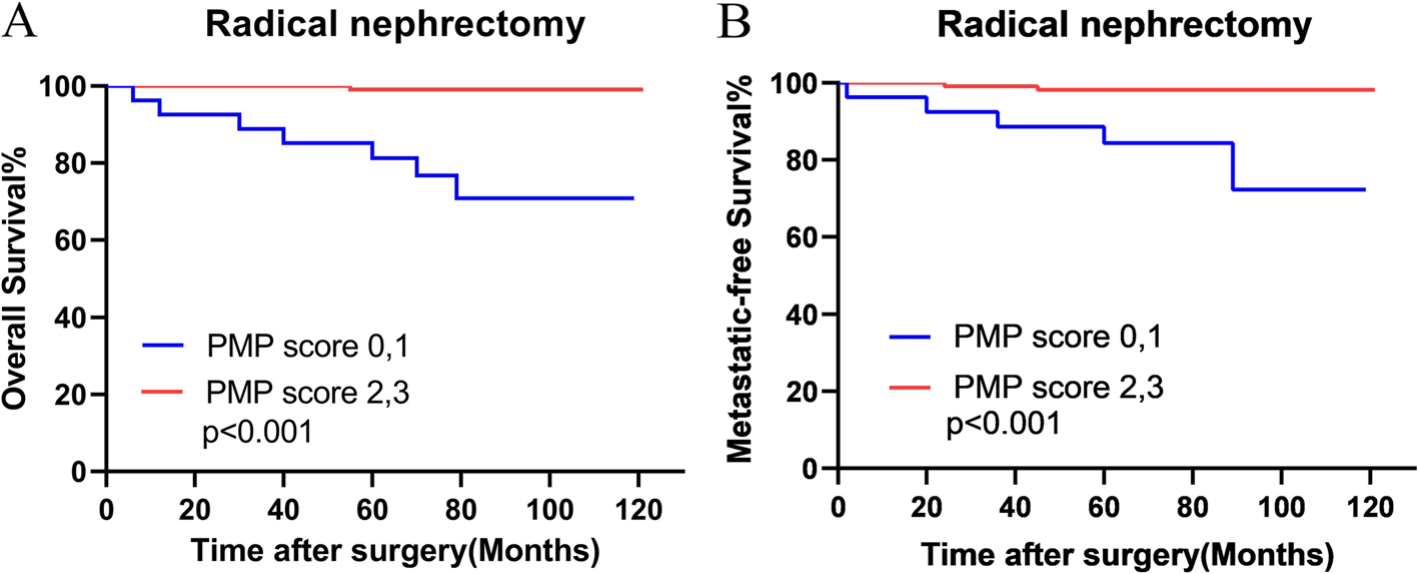
Kaplan–Meier analysis for overall survival (OS) (**A**) and metastatic-free survival (MFS) (**B**) of radical nephrectomy patient group with high and low risk of PNI-MLR-PLT scores. PMP score: PNI-MLR-PLT score

Surgical options may also make a difference in patients’ prognosis. Thus, we further assessed the outcomes of 140 patients with radical nephrectomy according to PNI. As Fig. 7 showed, patients with higher PNI-MLR-PLT score had significantly better OS (*P<* 0.001) and MFS (*P<*0.001) than patients with lower PNI-MLR-PLT score after radical nephrectomy (Fig. 7A and B).

### Cox regression analysis and predictive efficacy of PNI-MLR-PLT score

Next, Cox regression models were used to identify the independent risk factors of survival for patients with non-metastatic ccRCC. Univariable analysis revealed that six variables including age, anemia, tumor size, PNI-MLR-PLT score, pathologic T stage and tumor grade were significantly associated with OS (*P<* 0.05), and seven variables including hypertension, anemia, tumor size, PNI-MLR-PLT score, pathologic T stage and tumor grade, lymphatic and microvascular invasion were correlated to MFS (*P<* 0.05) (Table 4).

**Table 4:** Univariate analysis of parameters for the prediction of survival outcomes in 164 non-metastatic ccRCC patients

Parameters	OS		MFS	
	HR	P	HR	P
Gender	0.61(0.30–1.26)	0.181	1.19(0.27–5.31)	0.824
Age	1.18(1.08–1.30)	< 0.001	1.06(0.99–1.14)	0.085
Smoking	0.90(0.44–1.84)	0.768	1.56(0.35–6.99)	0.561
Drinking	0.73(0.33–1.64)	0.449	0.04(0.00–304.09)	0.476
Hypertension	0.82(0.41–1.64)	0.576	9.35(1.12–77.75)	0.039
DM	0.85(0.30–2.43)	0.765	1.82(0.22–15.11)	0.581
Anemia	0.16(0.03–0.81)	0.027	5.26(1.02–27.11)	0.047
Renal dysfunction	2.89(0.36–23.51)	0.321	0.05(0.00–1.06E)	0.723
ALT	0.99(0.94–1.04)	0.584	0.99(0.95–1.04)	0.727
AST	0.98(0.90–1.07)	0.657	0.90(0.77–1.05)	0.170
BUN	1.12(0.97–1.29)	0.132	1.10(0.91–1.33)	0.310
Tumor number	0.05(0.00–14,033,642.09)	0.760	0.05(0.00–2.67E)	0.767
Tumor size	13.33(3.33–53.35)	< 0.001	17.39(3.89–77.75)	< 0.001
PMP score (2 + 3/0 + 1)	0.07(0.01–0.34)	0.001	0.08(0.02–0.40)	0.002
T stage (III + IV/ I + II)	15.61(3.87–62.91)	< 0.001	20.80(4.63–93.38)	< 0.001
Grade (III + IV/I + II)	100.78(12.37–820.98)	< 0.001	100.91(12.05–845.06)	< 0.001
Tumor hemorrhage	0.05(0.00–9123.80)	0.618	0.04(0.00–15,007.24)	0.632
Tumor necrosis	0.05(0.00–420,311.62)	0.708	0.05(0.00–1.99E)	0.733
Lymphatic and microvascular infiltration	3.49(0.43–28.40)	0.242	9.76(1.89–50.32)	0.006

*ccRCC* Clear cell renal cell carcinoma, *DM* Diabetes mellitus, *BUN* Urea nitrogen, *ALT* Alanine aminotransferase, *AST* Glutamate aminotransferase, *PMP score* PNI-MLR-PLT score

Then, the multivariable analysis was performed and results showed that PNI-MLR-PLT score was independent protective factor for OS (HR= 0.106, 95% CI, 0.017-0.678, *P=* 0.018) and MFS (HR= 0.100, 95% CI, 0.011-0.927, *P=* 0.043) (Table 5). Besides, our data indicated that older age was one of the independent risk factor for OS (HR= 19.782, 95% CI, 1.551-252.318, *P=* 0.022). Higher T stage and tumor grade were independent risk factors for OS (T stage: HR= 4.655, 95% CI, 1.899-24.106, *P=* 0.027; tumor grade: HR= 39.445, 95% CI, 4.410-352.804, *P=* 0.001) and MFS (T stage: HR= 14.615, 95% CI, 1.297-164. 655, *P=* 0.030; tumor grade: HR= 56.498, 95% CI, 5.078-628.596, *P=* 0.001). Generally, our data demonstrated that high PNI-MLR-PLT score may be one of the protective factors of cancer prognosis for non-metastatic ccRCC patients.

**Table 5:** Multivariate analysis of parameters for the prediction of survival outcomes in 164 non-metastatic ccRCC patients

Parameters	OS		MFS	
	HR	P	HR	P
Age	19.78(1.55–252.32)	0.022	-	-
Hypertension	-	-	-	0.060
Anemia	-	0.222	-	0.888
Tumor size	-	0.824	-	0.295
PMP score (2 + 3/0 + 1)	0.11(0.02–0.68)	0.018	0.10(0.01–0.93)	0.043
T stage (III + IV/ I + II)	4.66(1.90–24.11)	0.027	14.62(1.30–164.66)	0.030
Grade (III + IV/I + II)	39.45(4.41–352.80)	0.001	56.50(5.08–628.60)	0.001
Lymphatic and microvascular infiltration	-	-	-	0.061

*ccRCC* Clear cell renal cell carcinoma, *PMP score* PNI-MLR-PLT score

## Discussion

Previous studies stated that systemic inflammation is closely related to tumorigenesis and development [14, 15]. Tumor-associated inflammation refers to the infiltration of inflammatory cells in cancer tissue and their secretion of tumor mediators [16]. The raveled inflammatory cytokines and their complex interactions are important components of tumor microenvironment, which have vital impacts on tumor recurrence and metastasis by influencing tumor growth, angiogenesis and tumor immune response [17]. Circulating white cells play pivotal roles in cancer immune responses [18]. Macrophages are considered to be pro-carcinogenesis and associated with poor outcomes. Whereas, circulating lymphocytes exhibit effective anti-tumor cellular immune response by secreting various cytokines such as interferon and tumor necrosis factor [19]. Thus, sufficient lymphocytes to a certain extent reflect the systemic immune defense capacity from cancer [20, 21]. Previous reports have shown that monocytes can directly kill tumor cells by producing IFN-αand antibody-dependent cellular cytotoxicity (ADCC), which mediate cancer cell apoptosis and death [22].

In addition, tumor cells promote the production and release of platelets by activating the IL-6, and excessive platelets increase the risk of vascular embolism in cancer patients [23]. The platelet vice versa promote tumor growth and invasion by releasing cytokines such as vascular endothelial growth factor (VEGF), platelet derived growth factor (PDGF) and transforming growth factor-β (TGF-β) [12, 23].

The nutritional and metabolic status of the body may equally correlated with cancer progression and metastasis [24, 25]. Serum albumin is specifically synthesized in the liver. Reduced serum albumin levels represent the state of malnutrition and reflect the body's ongoing systemic inflammatory response. Previous studies have shown that preoperative serum albumin levels is associated with human cancer survival, including renal cell carcinoma [26–28]. PNI covers lymphocytes and serum albumin, and reflects both immune homeostasis and nutritional metabolism of the body.

In the present study, we focuses on the significance of PNI, MLR and PLT on the postoperative clinical outcomes in patients with non-metastatic ccRCC. In line with previous findings [7–9, 29], our data suggested that all these three indicators are associated with patients’ postoperative survival. For the first time, we developed a new model combining PNI, MLR and PLT, and investigated its prognostic value. Our results showed that preoperative PNI-MLR-PLT score level decreased with the rise of pathological T stage and tumor grade. Through Pearson’s chi-square analysis, PNI-MLR-PLT score were found correlated with anemia, renal dysfunction, PLT, MLR, PNI, tumor size, pathologic T stage and tumor grade, lymphatic and microvascular infiltration. Also, significantly better OS and MFS were observed in patients with higher PNI-MLR-PLT score compared to those with lower PNI-MLR-PLT score. Moreover, Cox regression analysis indicated that high PNI-MLR-PLT score was an independent protective factor for cancer survival in patients with non-metastatic ccRCC. In addition, we further stratified the enrolled patients based on pathological T stage and tumor grade, and then performed a subgroup analysis and gained consistent results. The difference was not statistically significant in T stage 3+4 (MFS) and tumor grade 3+4 (OS, MFS) subgroups, which we consume was attributed to the limited number of subgroup cases. Surgical options including radical or partial nephrectomy may also affects the clinical outcomes of patients. Thus, we further assessed the prognostic significance of PNI-MLR-PLT score in 140 patients with radical nephrectomy and obtained same results. As for the partial nephrectomy subgroup, however, no further analysis was performed since no death, relapse or metastasis occurred in these patients. A larger cases of study is needed to validate the results in the future. However, based on the convincing data displayed in our study, it is still reasonable to state that PNI-MLR-PLT score could serve as a reliable and low-cost indicator for the prediction of postoperative survival in non-metastatic ccRCC patients.

There are limitations in this study. Firstly, this is a retrospective study with a small sample size, which may be subject to selection bias and interference by other uncharted factors. Secondly, the changes of PNI-MLR-PLT score after treatment were not monitored and the potential significance was not elucidated. Thirdly, we only enrolled patients with non-metastatic ccRCC, and the results are not applicable to all renal cancer patients.

## Conclusion

High PNI-MLR-PLT score was associated with better survival in patients with non-metastatic ccRCC. PNI-MLR-PLT score may serve as a convenient and reliable indicator for the prediction of postoperative outcomes.

## Data Availability

The original data of the present study were available from the corresponding author on reasonable requests.

## Abbreviations

AJCC: American Joint Committee on Cancer
AUC: Area under ROC curve
ccRCC: Clear cell renal cell carcinoma
HR: Hazard ratio
MFS: Metastasis-free survival
MLR: Monocyte-to-lymphocyte ratio
OS: Overall survival
PNI: Prognostic nutritional index
RCC: Renal cell carcinoma
ROC: Relative operating characteristic
